# Densely packed needles along the shoots of evergreen conifers exhibit shade-acclimated photosynthetic characteristics even under full sunlight

**DOI:** 10.1093/aob/mcag030

**Published:** 2026-04-08

**Authors:** Mitsutoshi Kitao, Kenichi Yazaki, Tetsuto Sugai, Hisanori Harayama, Evgenios Agathokleous, Ryouichi Tanaka

**Affiliations:** Hokkaido Research Center, Forestry and Forest Products Research Institute, Sapporo 062-8516, Japan; Hokkaido Research Center, Forestry and Forest Products Research Institute, Sapporo 062-8516, Japan; Hokkaido Research Center, Forestry and Forest Products Research Institute, Sapporo 062-8516, Japan; Department of Plant Ecology, Forestry and Forest Products Research Institute, Tsukuba 305-8687, Japan; Department of Ecology, School of Ecology and Applied Meteorology, Nanjing University of Information Science & Technology (NUIST), Nanjing 210044, China; Institute of Low Temperature Science, Hokkaido University, Sapporo 060-0819, Japan

**Keywords:** Mutual shading, needle configuration, needle longevity, photosynthetic light response, shade tolerance

## Abstract

**Background and Aims:**

Evergreen conifer shoots that develop under full sunlight eventually move to the shade within the canopy as the tree grows. We hypothesized that densely packed needles develop shade characteristics even under full sunlight due to mutual shading, allowing them to survive longer in the canopy shade.

**Methods:**

To test this hypothesis, we measured photosynthetic light responses at the shoot and needle levels in four species: *Picea glehnii* (shade-intolerant), *P. jezoensis* (intermediate shade-tolerant), *Abies sachalinensis* (shade-tolerant) and *Taxus cuspidata* (highly shade-tolerant), grown under open (100 % sunlight) and shade (10 % sunlight) conditions.

**Key Results:**

In *P. glehnii*, *P. jezoensis* and *A. sachalinensis*, needle-level photosynthetic and morphological traits showed minimal differences between light conditions, indicating the development of shade-characteristic needles even under full sunlight. In contrast, open-grown needles of *T. cuspidata* exhibited sun-acclimated traits, including higher photosynthetic capacity and thicker mesophyll tissue than shade-grown counterparts. Shoot-level photosynthetic capacity was higher in more shade-intolerant species with denser needle packing, and in open-grown seedlings across species.

**Conclusions:**

Shoot-level acclimation to light environments in *P. glehnii*, *P. jezoensis* and *A. sachalinensis* was governed primarily by needle configuration. In *T. cuspidata*, it was influenced by both needle-level photosynthesis and shoot morphology. The presence of shade-characteristic needles along with reduced needle packing from age-related stem thickening may contribute to the longevity of evergreen conifer shoots under canopy shading.

## INTRODUCTION

One of the most notable features of evergreen conifer trees is the longevity of their needles. These needles can typically survive for 4–5 years, and in some cases up to 10 years or more. During their lifespan, conifer needles develop under open conditions, receiving high solar irradiation at the outer edges of the canopy. As the tree grows, these needles are gradually pushed inward towards the canopy with lower light availability due to within-canopy shading. Consequently, conifer needles inevitably experience a significant change in their light environment, transitioning from open, sunlit conditions to deep shade over the course of their life. Old needles of conifer trees retain a considerable amount of nitrogen and act as a source of nitrogen, rather than photosynthates, due to their lower photosynthetic capacity, when new shoots develop in spring (Wyka *et al*., 2016; Kitao *et al*., 2019*a*). Thus, survival of old needles within the canopy is essential for conifer growth to keep a buffer of nitrogen.

Light acclimation of photosynthesis is crucial for plant growth and survival, particularly within a canopy and in the forest understorey. For example, Japanese oak (*Quercus mongolica*), a deciduous broadleaved tree species, developed under low light conditions exhibits a lower capacity for photosynthesis and respiration, along with a lower leaf mass per area (LMA). In contrast, leaves developed under high light conditions have a higher capacity for photosynthesis and respiration, as well as a greater LMA (Kitao *et al*., 2012). Such an acclimation to light environments within a canopy is broadly observed both in broadleaved and conifer tree species (e.g. Bond *et al*., 1999; Koike *et al*., 2001). Thus, leaf morphology plays a significant role in determining light acclimation, where shade-tolerant species are capable of developing thinner leaves for acclimation to deeper shade (Kitao *et al*., 2006). However, once leaves mature, their morphology becomes less flexible, limiting their ability to acclimate to changes in light conditions (Oguchi *et al*., 2003, 2005; Niinemets *et al*., 2004; Niinemets, 2007). It remains an open question how conifer needles, initially developed under sunlight, manage to survive for extended periods under low light conditions.

Leaf angle is a relevant factor for photosynthetic performance and circumvention of photoinhibition at the leaf level, influencing light interception at the leaf surface (Yang *et al*., 2023). Similarly, branching pattern (i.e. canopy architecture) of tree species determines the overall photosynthetic performance of a canopy (Kull and Tulva, 2002; Mediavilla and Escudero, 2023). Additionally, in conifer trees, needle configuration along a shoot can significantly impact photosynthetic performance at the shoot level. Needle configuration varies widely among conifer species. For example, the needles of spruces (*Picea* species) radiate outwards from the shoots, forming a dense and three-dimensional foliage structure. In firs (*Abies* species), the needles exhibit a spiral arrangement along the shoots, producing a comb-like appearance. Pines (*Pinus* species), on the other hand, have a relatively sparse packing of needles within a shoot, with each needle arranged in bundles called fascicles. The needles of hemlocks (*Tsuga* species) and yews (*Taxus* species) are horizontally attached to the shoots, leading to a less densely packed configuration. In addition to this interspecific variation, needle packing and configuration are influenced by light conditions. For instance, in the shade-tolerant spruce species *Picea abies*, shoots grown under deeper shade conditions within a canopy exhibit sparser needle packing, characterized by a lower ratio of total needle area to projected needle area (*A*_T_/*A*_P_) (Niinemets, 1997, 2010). This adaptation enhances light interception at the individual needle level in low-light environments. Conversely, in sun-exposed shoots, a higher *A*_T_/*A*_P_ ratio indicates that mutual shading among densely packed needles results in relatively low light intensities at the needle level, even under strong sunlight.

Here, we hypothesize that densely packed needles in sun-exposed shoots might possess characteristics of shade-acclimated photosynthesis. This adaptation could be advantageous for efficient photosynthesis and survival under shade, as sun-exposed shoots are gradually positioned inside the canopy with shoot age. This process, combined with the thinning out of needles (resulting in a lower *A*_T_/*A*_P_ ratio) (Niinemets, 1997, 2010), confers shoot-level shade tolerance in some conifer species. To test this hypothesis, we measured photosynthetic light responses at both the shoot and needle levels in four evergreen coniferous species native to northern Japan (*Picea glehnii*, *Picea jezoensis*, *Abies sachalinensis* and *Taxus cuspidata*), each with distinct shade tolerance and needle configuration.

## MATERIALS AND METHODS

### Plant materials

We used four evergreen conifer species native to northern Japan, Hokkaido, each with distinct shade tolerance: Sakhalin spruce (*Picea glehnii*), Ezo spruce (*P. jezoensis*), Sakhalin fir (*Abies sachalinensis*) and Japanese yew (*Taxus cuspidata*). *Picea glehnii* is classified as a shade-intolerant pioneer species, often found in harsh habitats, such as serpentine soils (Nakata and Kojima, 1987; Kayama *et al*., 2006). *Picea jezoensis* and *A. sachalinensis* are classified as shade-tolerant species whose seedlings are found in the forest understorey, with *A. sachalinensis* exhibiting greater shade tolerance than *P. jezoensis* (Iijima *et al*., 2009). *Taxus cuspidata* is also a shade-tolerant species, thriving in deeper shade conditions where *A. sachalinensis* cannot survive. In this study, we ranked shade tolerance as *T. cuspidata* > *A. sachalinensis* > *P. jezoensis* > *P. glehnii*.

Four-year-old seedlings of *T. cuspidata* (approximately 35 cm in height), obtained from a commercial nursery, were transplanted into 4-L plastic pots filled with a mixture of clay loam soil and Kanuma pumice soil (1:1 by volume) at the beginning of July 2021. Five-year-old seedlings of *P. glehnii* were transplanted at the end of April 2022, and 5-year-old seedlings of *P. jezoensis* and *A. sachalinensis* were transplanted at the end of April 2023. The latter three species were obtained from the nursery of Hokkaido Research Center, Forestry and Forest Products Research Institute (43.0°N, 141.4°E; 180 m a.s.l.), with an initial height of approximately 20 cm. We added 40 g of commonly used fertilizer (Osmocote Exact Standard 15-9-11 +TE, HYPONeX Japan, Osaka, Japan) to each pot. Shade treatment (relative irradiance ≈ 10 %) was conducted using four frames (width: 60 cm × length: 95 cm × height: 105 cm) covered with shade cloths, set in an open site at the Hokkaido Research Center. Three seedlings per species were placed in each shade frame (totalling 12 seedlings per species in the shade treatment) immediately after their transplantation into the pots (shade treatment). Another 12 seedlings per species were placed in open natural light conditions (open treatment). Gas exchange measurements were conducted from the end of September to the beginning of October, 2024, when the seedlings were 7 years old for *T. cuspidata* (light treatment: July 2021 to October 2024, ≈50 cm in height) and *P. glehnii* (April 2022 to October 2024, ≈35 cm), and 6 years old for *P. jezoensis* (April 2023 to October 2024, ≈30 cm) and *A. sachalinensis* (April 2023 to October 2024, ≈30 cm).

### Gas exchange measurements

Photosynthetic light responses were measured on current-year shoots that flushed in spring 2024. These shoots developed entirely under the light conditions (open or shade) to which the plants were assigned. Measurements were conducted after the shoots had fully matured (approximately 4 months old) (Wyka *et al*., 2016), from late September to early October 2024, when the average daily maximum temperature was 21.4 °C. Net photosynthetic rate (*P*_n_) was measured with a portable photosynthesis system (Li-6400, Li-Cor, Lincoln, NE, USA) combined with a conifer leaf chamber (Li-6400-22, Li-Cor) and a light source (Li-6400-18, Li-Cor). Measurements were conducted at an ambient CO_2_ concentration of 400 µmol mol^−1^, various light intensities (photosynthetically active radiation of 0, 50, 100, 200, 400, 600, 800, 1000 and 1500 µmol m^−2^ s^−1^), and a leaf temperature of 25 °C. First, we measured photosynthesis of a whole shoot (shoot-level photosynthesis). Then, we removed upper and lower needles, and kept horizontally attached needles without overlapping (cf. Carter and Smith, 1985). We measured photosynthesis of one-layered needles and the stem. Finally, we measured respiration rate of the stem after removing all needles. This series of procedures allowed us to determine shoot-level photosynthesis based on the projected area of shoot (*P*_shoot_), and needle-level photosynthesis based on the needle area (*P*_needle_). Details are shown in Supplementary Data Fig. S1. We considered the influence of wound respiration induced by needle detachment to be negligible for the estimation of needle respiration (cf. Supplementary Discussion). Note that the conventional needle-level photosynthesis, determined as whole shoot gas exchange divided by total needle area, is not suitable to assess the needle-level photosynthetic light response, since the light intensity received at the surface of needles is attenuated by mutual shading. The procedure used in the present study is essential for an accurate assessment of photosynthetic light responses at the needle level.

### Chlorophyll fluorescence measurements

*F*_v_/*F*_m_ = (*F*_m_ − *F*_o_)/*F*_m_, was measured in current-year shoots after an overnight dark-adaptation with a portable chlorophyll fluorometer (PAM-2000, Walz, Effeltrich, Germany), where *F*_m_ is the maximum fluorescence level elicited by a pulse of saturating light (≈10 000 µmol m^−2^ s^−1^), and *F*_o_ is the minimum fluorescence level. Seedlings were transferred to the laboratory in the evening prior to the *F*_v_/*F*_m_ measurements. The following morning, after an overnight dark adaptation, the *F*_v_/*F*_m_ measurements were conducted. As *F*_v_/*F*_m_ was recently found to be uncorrelated with the efficiency of photosystem II (PSII) photochemistry (Sipka *et al*., 2021), we considered *F*_v_/*F*_m_ as an empirical measure of PSII activity, and declines in overnight dark-adapted *F*_v_/*F*_m_ as an empirical measure of photoinhibition (Werner *et al*., 2001; Kitao *et al*., 2024).

### Needle packing within a shoot

As a measure of needle packing along a shoot, we used the ratio of total needle area to projected shoot area (*A*_T_/*A*_P_) (Niinemets, 1997, 2010). Photographs of a shoot with a scale placed in the open chamber (Li-6400-22) were taken by a digital camera (E-5, Olympus, Tokyo, Japan) for the determination of projected shoot area. Horizontally attached needles, used for the measurement of needle-level photosynthesis, were all detached and scanned by a scanner (CanoScan LIDE210, Canon, Tokyo, Japan) for needle area determination. Area was estimated by using image processing software (LIA32, v.0.377e, Kazukiyo Yamamoto, 2004, https://www.agr.nagoya-u.ac.jp/∼shinkan/LIA32/index-e.html). After the measurements of gas exchange, sampled needles were dried at 70 °C to constant weight in an electric oven. LMA was calculated based on the needle area and needle dry mass. Total needle area was estimated as *A*_T_ = [total needle dry mass]/LMA.

### Needle nitrogen content

Dry-mass-based needle nitrogen content (N_mass_) was determined by a nitrogen carbon analyser with oxygen circulating combustion system (SUMIGRAPH, NC 22F, Sumika Chem. Anal. Service, Osaka, Japan). Area-based needle nitrogen content (N_area_) was estimated as N_area_ = N_mass_ × LMA.

### Microscopic analysis for needle anatomy

Three to four needles from the current-year shoots were sampled from four individuals in each species using a sharp blade and fixed overnight with a 4 % glutaraldehyde fixing solution diluted in phosphate buffer (pH = 7.2). The samples were dehydrated in a stepwise ethanol series and embedded in epoxy resin (Epon 812, TAAB Laboratories Equipment Ltd, UK) with *n*-butyl glycidyl ether (QY-1) as a substituent. Transverse sections of 1.5-µm thickness were cut using a rotary microtome (HM340E, PHC Holdings Corp., Japan) equipped with a glass knife. Semi-thin sections were stained with 0.05 % toluidine blue aqueous containing 0.3 % boric acid and observed using an optical microscope (ECLIPSE Ni, Nikon, Co, Ltd, Japan).

To characterize needle morphological traits, we quantified three parameters related to mesophyll tissue based on transverse needle cross-sections (Supplementary Data Figs S2–S5), each of which is considered relevant to photosynthetic performance:

1. average needle thickness (*T*_avg_): calculated as the total cross-sectional area divided by needle width;
2. average mesophyll thickness based on needle width (*T*_mes.w_): calculated as the total mesophyll area divided by needle width; and
3. fraction of mesophyll parenchyma (*f*_mes_): defined as the ratio of mesophyll area to total cross-sectional area.

**Fig. 1.**
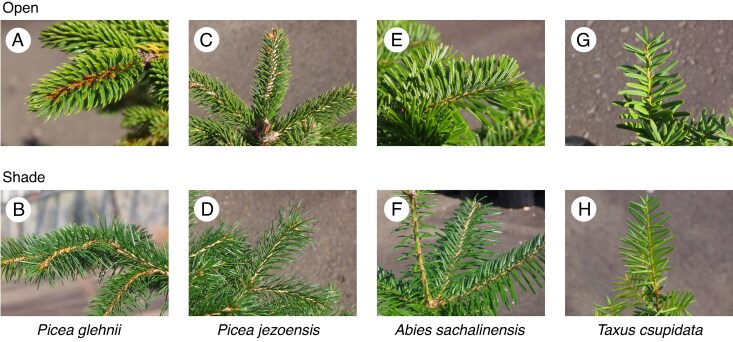
Needle appearance in the shoots of four coniferous species grown under open (upper panels) and shade (lower panels) conditions: *Picea glehnii* (A, B), *P. jezoensis* (C, D), *Abies sachalinensis* (E, F) and *Taxus cuspidata* (G, H).

For gas exchange measurements in one-layer needles of *P. glehnii* and *P. jezoensis* (cf. Supplementary Data Fig. S1), the needles were attached horizontally to shoots with a 90° rotation (cf. Fig. 1, and Figs S2 and S3). Accordingly, vertical width was used in the calculation of both *T*_avg_ and *T*_mes.w_ for these species.

### Light attenuation due to needle packing in conifer shoots

The response of photosynthesis to incident irradiance was modelled using the convexity equation (Leverenz *et al*., 1990):


$$\theta (P_{n}+R_{n}{)}^{2}-(\phi \mathrm{PPFD}+P_{\max})(P_{n}+R_{n})+\phi \mathrm{PPFD}*P_{\max}=0,$$

where *P*_n_ is the net photosynthetic rate at a given photosynthetic photon flux density (PPFD), *R*_n_ is the dark respiration rate at 25 °C, ϕ is the initial slope (maximum quantum yield), *P*_max_ is the light-saturated photosynthetic rate and θ is the convexity parameter of the light-response curve.

Shoot-level parameters, *P*_max.shoot_, *R*_n.shoot_, θ_shoot_ and ϕ_shoot_, were derived from light-response curves based on shoot projected area. Similarly, needle-level parameters, *P*_max.needle_, *R*_n.needle_, θ_needle_ and ϕ_needle_, were obtained from curves based on needle surface area.

Gross photosynthetic rate (*P*_g_) was calculated as *P*_g_ = *P*_n_ + *R*_n_, yielding the following expression:


$$\begin{array}{rl} P_{g} & =(\phi \;\mathrm{PPFD}+P\max-({\left(\phi \mathrm{PPFD}+P\max\right)}^{2}\\ & \;{-4\;\theta \;\phi \;\mathrm{PPFD}\;P\max)}^{0.5})/\left(2\theta \right) \end{array}.$$

Shoot-level gross photosynthesis (*P*_g.shoot_) was modelled as a scaled needle-level response, where τ denotes the theoretical shoot transmittance coefficient, the fraction of incident light reaching needle surfaces. The ratio (*P*_max.shoot_)/(*P*_max.needle_) scales the needle-level response to reflect shoot-level photosynthetic capacity. Assuming needle-level gross photosynthesis is defined as *P*_g.needle_ = *f*(PPFD), shoot-level gross photosynthesis can be expressed as:


$$P_{g.\mathrm{shoot}}=(P_{\max.\mathrm{shoot}}/P_{\max.\mathrm{needle}})f(\tau \mathrm{PPFD}).$$

Given that ϕ, θ, and *P*_max_ are available at both shoot and needle scales, τ can be estimated as a theoretical proxy for internal light attenuation caused by needle packing.

### Statistical analyses

A Tukey–Kramer post-hoc test was employed to investigate the differences in morphological and physiological traits among the combinations of species and light conditions (R Core Team, 2023). We applied linear regression analyses to investigate the effects of LMA, N_area_ and *A*_T_/*A*_P_ on photosynthetic traits, setting the former as the independent variables, and photosynthetic traits as the dependent variables. The level of significance was set at *α* = 0.05.

## RESULTS

### Needle arrangement within a shoot

**Fig. 2.**
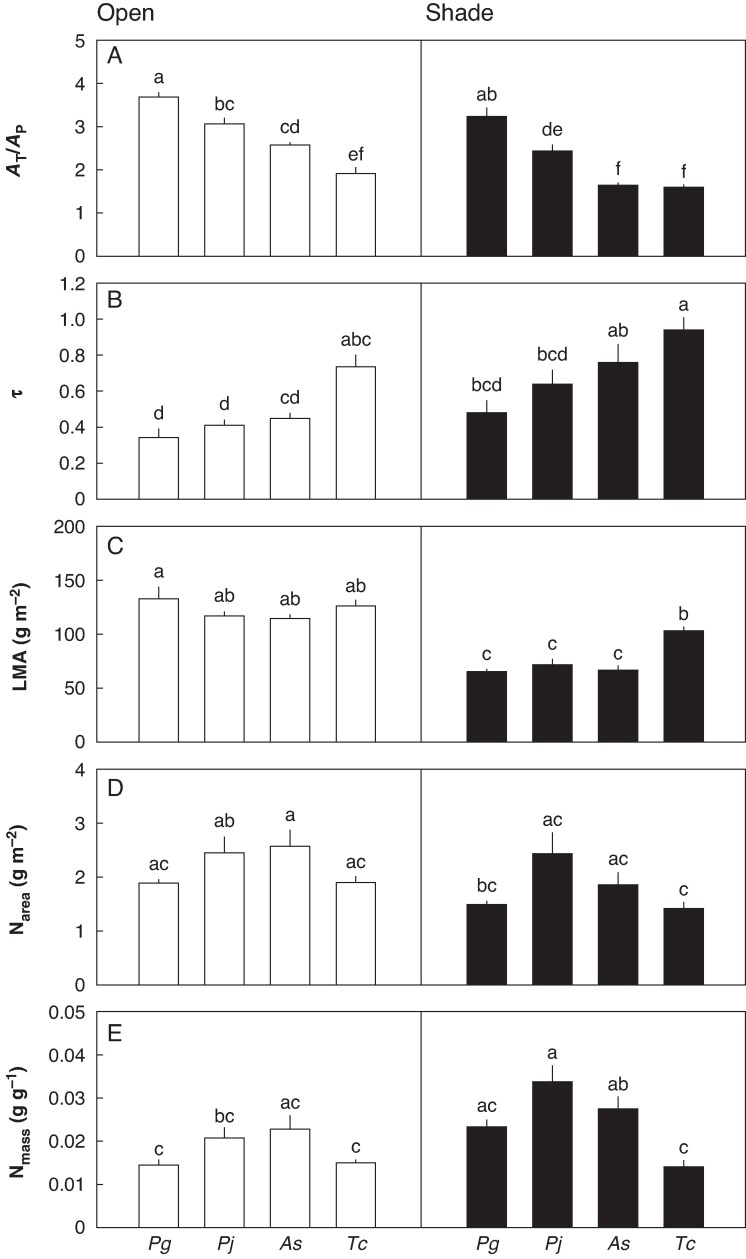
Ratio of total to projected area (*A*_T_/*A*_P_) in shoots (A), shoot transmittance coefficient (τ, B), leaf mass per area (LMA) in needles (C), dry mass-based needle N (N_mass_, D) and area-based needle N (N_area_, E) of four evergreen conifer species (*Picea glehnii*: *Pg*, *P. jezoensis*: *Pj*, *Abies sachalinensis*: *As*, and *Taxus cuspidata*: *Tc*) grown under open (left) and shade conditions (right panels). Different letters indicate significant differences among means of the combination of species and light conditions at *P* < 0.05. Values are means + s.e. (*n* = 4).

Based on observations, shade-tolerant species tended to have shoots with sparsely attached needles. Additionally, shade-grown seedlings exhibited less densely packed needles compared to sun-grown seedlings (Fig. 1). A measure of needle packing within a shoot (*A*_T_/*A*_P_) showed higher values in *P. glehnii* > *P. jezoensis* > *A. sachalinensis* > *T. cuspidata*, with commonly higher *A*_T_/*A*_P_ observed in open-grown seedlings (Fig. 2A).

### Theoretical shoot transmittance coefficient

**Fig. 3.**
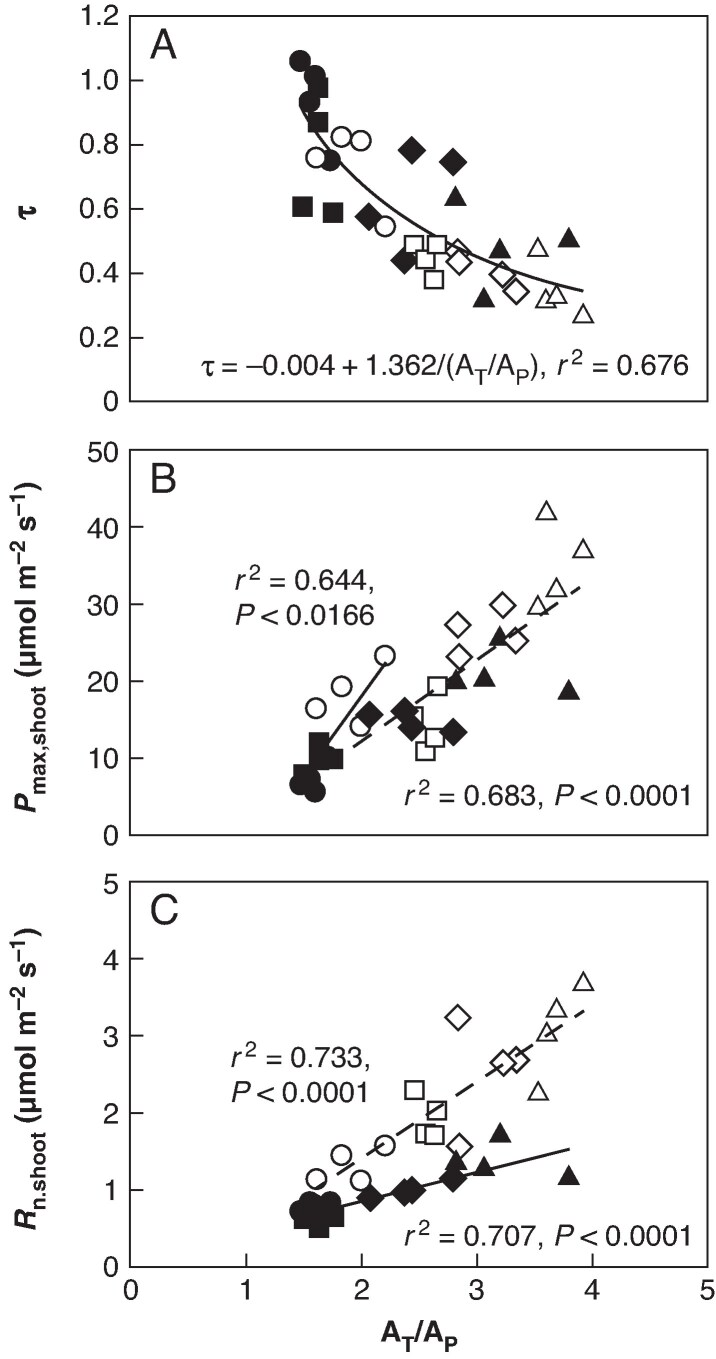
Relationships between the ratio of total to projected shoot area (*A*_T_/*A*_P_) and three shoot-level physiological parameters in four conifer species – *Picea glehnii* (triangles), *P. jezoensis* (diamonds), *Abies sachalinensis* (rectangles) and *Taxus cuspidata* (circles) – grown under open (open symbols) and shade (closed symbols) conditions. (A) Relationship between *A*_T_/*A*_P_ and the shoot transmittance coefficient due to needle packing (τ). The fitted curve is described by the equation: τ = −0.004 + 1.362/(*A*_T_/*A*_P_), with *r*^2^ = 0.676. (B) Relationship between *A*_T_/*A*_P_ and shoot-level maximum photosynthetic rate (*P*_max.shoot_). Dashed line represents a linear regression for pooled data of *P. glehnii*, *P. jezoensis* and *A. sachalinensis* across light conditions (*n* = 24). Solid line indicates a linear regression for open- and shade-grown seedlings of *T. cuspidata* (*n* = 8). (C) Relationship between *A*_T_/*A*_P_ and shoot-level respiration rate (*R*_n.shoot_). Dashed line represents a linear regression for pooled data of open-grown seedlings across species (*n* = 12), and solid line represents shade-grown seedlings (*n* = 12).

*Taxus cuspidata* exhibited significantly higher τ values than *P. glehnii* and *P. jezoensis* under both open and shade conditions, whereas no significant differences were detected between *T. cuspidata* and *A. sachalinensis* (Fig. 2B). In general, shade-grown seedlings showed higher τ than their open-grown counterparts, although a statistically significant difference between light conditions was observed only in *A. sachalinensis*. Across species and light environments, τ was inversely proportional to the shoot area ratio *A*_T_/*A*_P_ (Fig. 3A).

### Leaf mass per area of a needle

Significantly higher LMA of needles was observed in open-grown seedlings compared to shade-grown seedlings, except for *T. cuspidata* (Fig. 2C). There was no difference in LMA among species under open conditions, whereas higher LMA was observed in *T. cuspidata* than in the other three species under shade conditions.

### Leaf N content

Area-based leaf N content (N_area_) showed no significant difference among species within each light condition, and no difference between light conditions within each species (Fig. 2D). Regarding dry mass-based leaf N content (N_mass_), no difference was observed among species in the open condition, while higher N_mass_ was observed in *P. jezoensis* and *A. sachalinensis* than in *T. cuspidata* in the shade condition (Fig. 2E). Between light conditions, open-grown seedlings of *P. jezoensis* showed significantly lower N_mass_ than the shade-grown counterparts.

### Photosynthetic traits

**Fig. 4.**
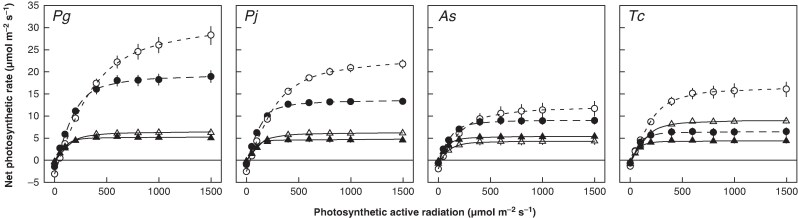
Light response curves at the shoot (circles) and needle (triangles) levels in the seedlings of four evergreen conifer species (*Picea glehnii*: *Pg*, *P. jezoensis*: *Pj*, *Abies sachalinensis*: *As*, and *Taxus cuspidata*: *Tc*) grown under open (open symbols) and shade (closed symbols) conditions. Measurements were conducted under an ambient CO_2_ of 400 µmol mol^−1^ and a leaf temperature of 25 °C. Values are means ± s.e. (*n* = 4). The responses of photosynthesis to incident irradiance were revealed by the convexity equations (dashed curves for shoot-level responses, and solid lines for needle-level responses).

**Fig. 5.**
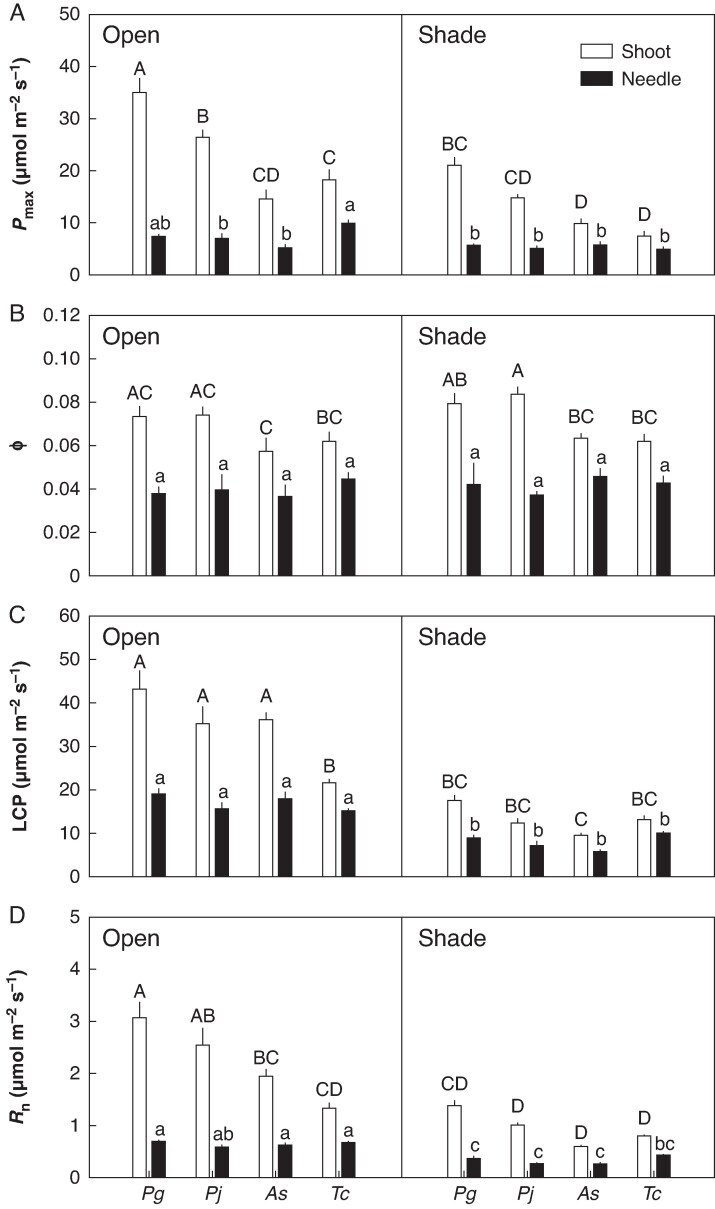
Maximum rate of photosynthesis (*P*_max_, A), initial slope (ϕ, B), light compensation point (LCP, C) and respiration rate (*R*_n_, D) at the shoot (open bars) and the needle level (solid bars) of four evergreen conifer species (*Picea glehnii*: *Pg*, *P. jezoensis*: *Pj*, *Abies sachalinensis*: *As*, and *Taxus cuspidata*: *Tc*) grown under open (left panel) and shade (right panel) conditions. Different letters indicate significant differences among means of the combination of species and light conditions at *P* < 0.05; capital letters for shoot responses, and lower-case letters for needle responses, respectively. Values are means + s.e. (*n* = 4).

**Fig. 6.**
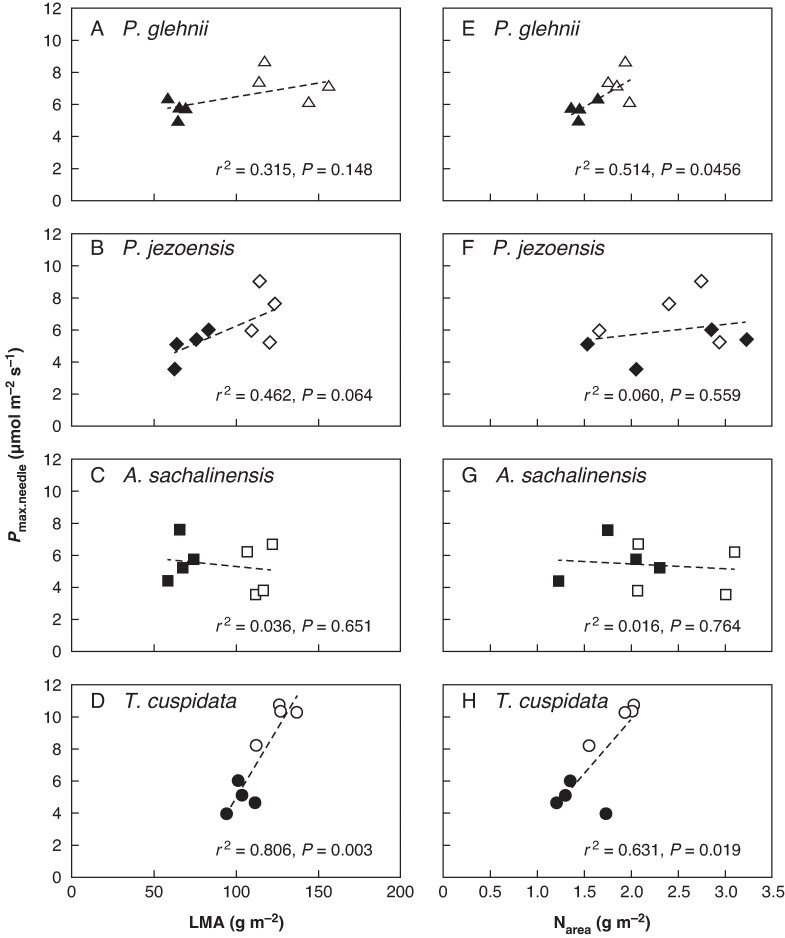
Relationship between leaf mass per area (LMA, left panels) and area-based leaf N concentration (N_area_, right panels), and needle-level maximum photosynthetic rate (*P*_max.needle_) in four conifer species (*Picea glehnii*: A, E, *P. jezoensis*: B, F, *Abies sachalinensis*: C, G, *Taxus cuspidata*: D, H) grown under open (open symbols) and shade (closed symbols) conditions. Dashed lines are linear regressions conducted for each species including both open- and shade-grown seedlings.

Photosynthetic traits were derived from light response curves at the shoot and needle levels (Fig. 4). Shoot-level maximum photosynthetic rate (*P*_max.shoot_) was higher in open-grown seedlings than in shade-grown seedlings (Fig. 5A). Among open-grown seedlings, *P. glehnii* showed the highest *P*_max.shoot_, and *P. jezoensis* had the second highest *P*_max.shoot_. Among shade-grown seedlings, higher *P*_max.shoot_ was observed in *P. glehnii* than in the other species, although the difference from *P. jezoensis* was non-significant. Except for open-grown *T. cuspidata*, similar values of needle-level maximum photosynthetic rate (*P*_max.needle_) were observed among species irrespective of light conditions. Higher *P*_max.needle_ was observed in open-grown *T. cuspidata* than in other combination, albeit the difference from *P. glehnii* was non-significant (Fig. 5A). Regarding *P*_max.needle_, higher LMA in open-grown seedlings than in shade-grown seedlings of *P. glehnii*, *P. jezoensis* and *A. sachalinensis* might not contribute to increasing *P*_max.needle_, while commonly higher *P*_max.needle_ was observed in *T. cuspidata* along with an associated higher LMA across light conditions (Fig. 6, left panels). Conversely, *P*_max.needle_ was positively correlated with N_area_ in *P. glehnii* and *T. cuspidata* across light conditions (Fig. 6, right panels). As *P*_max.needle_ showed no significant differences across species and light conditions, except for open-grown *T. cuspidata* (Fig. 5A), the variation in *P*_max.shoot_ can be explained by *A*_T_/*A*_P_, except in the case of open-grown *T. cuspidata* (Fig. 3B). The relatively higher *P*_max.needle_ in open-grown seedlings of *T. cuspidata* resulted in higher *P*_max.shoot_ at a given *A*_T_/*A*_P_.

The shoot-level initial slope of the photosynthetic light–response curve (ϕ_shoot_) showed no difference among species in open-grown seedlings, while higher ϕ_shoot_ was observed in seedlings of *P. jezoensis* than *A. sachalinensis* and *T. cuspidata*, but not *P. glehnii*, when grown under shade (Fig. 5B). Conversely, there was no difference in ϕ_needle_ among the combinations of species and light conditions. There was no difference in ϕ_shoot_ and ϕ_needle_ between open and shade conditions in all species.

The shoot-level light compensation point (LCP_shoot_) in open-grown seedlings of *T. cuspidata* was significantly lower than in those of the other three species, while there was no significant difference in LCP_shoot_ among shade-grown seedlings of the four species (Fig. 5C). Higher (*P* < 0.05) LCP_shoot_ was observed in open-grown seedlings of *P. glehnii*, *P. jezoensis* and *A. sachalinensis* compared to their counterparts in shade, whereas no difference (*P* > 0.05) was observed between open- and shade-grown seedlings of *T. cuspidata*. Needle-level LCP (LCP_needle_) showed no difference (*P* > 0.05) within each light condition, but open-grown seedlings had significantly higher LCP_needle_ than shade-grown seedlings regardless of species.

Needle-level respiration rate (*R*_n.needle_) showed no difference (*P* > 0.05) among species within each light condition (open or shade), whereas lower *R*_n.needle_ (*P* < 0.05) was observed in shade-grown seedlings than open-grown seedlings within each species (Fig. 4D). Higher *R*_n.needle_ was positively correlated with higher LMA irrespective of species and light conditions (Supplementary Data Fig. S6). Shoot-level respiration rate (*R*_n.shoot_) was influenced both by *R*_n.needle_ and *A*_T_/*A*_P_, where shade-grown seedlings showed lower *R*_n.shoot_ at a given *A*_T_/*A*_P_ (Fig. 3C).

### Overnight dark-adapted *F*_v_/*F*_m_

**Fig. 7.**
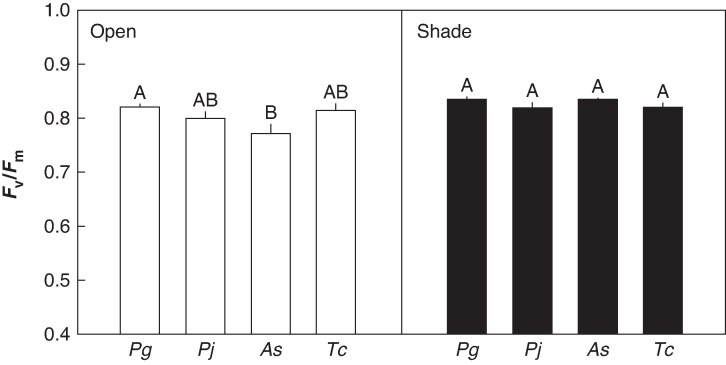
Overnight dark-adapted *F*_v_/*F*_m_ in shoots of four evergreen conifer species (*Picea glehnii*: *Pg*, *P. jezoensis*: *Pj*, *Abies sachalinensis*: *As*, and *Taxus cuspidata*: *Tc*) grown under open (left panel) and shade (right panel) conditions. Different letters indicate significant difference among means of the combination of species and light conditions at *P* < 0.05. Values are means + s.e. (*n* = 4).

Lower overnight dark-adapted *F*_v_/*F*_m_ was observed in open-grown seedlings of *A. sachalinensis* among all combinations of species and light conditions, although the difference from *P. jezoensis* and *T. cuspidata* in fully open conditions was non-significant (Fig. 7).

### Leaf anatomy

**Fig. 8.**
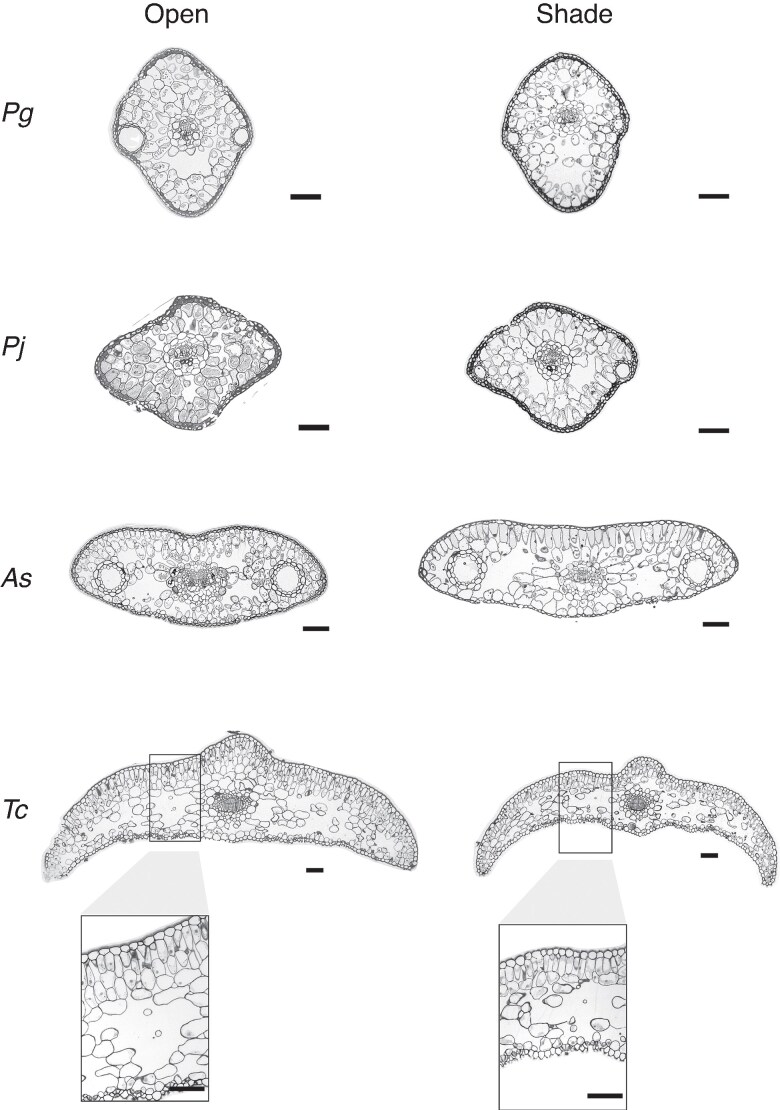
Transverse sections of the needles of four evergreen conifer species (*Picea glehnii*: *Pg*, *P. jezoensis*: *Pj*, *Abies sachalinensis*: *As*, and *Taxus cuspidata*: *Tc*) grown under open (left panel) and shade (right panel) conditions. Typical images among four individuals in each species grown under each light condition are shown (see Supplementary Data Figs S2–5). All images, except for the overview images of *Tc*, were acquired using a 10× objective lens, and the overview images of *Tc* were acquired using a 4× objective lens. In *Tc*, each photograph in the box shows a magnified image (10× objective lens). Bars = 100 µm.

**Fig. 9.**
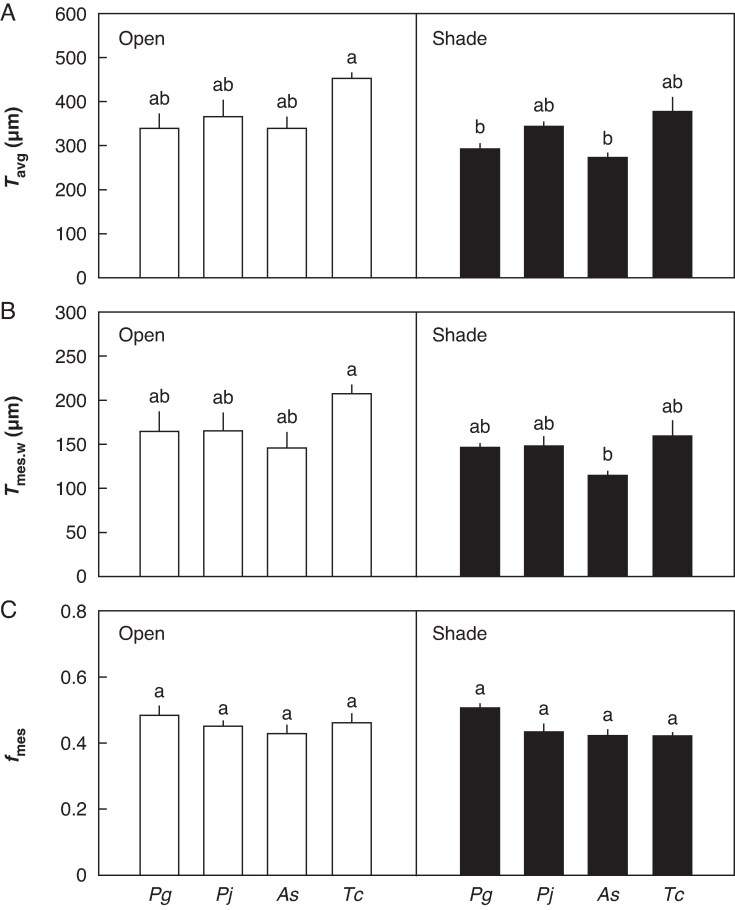
Average needle thickness (*T*_avg_) (A), average mesophyll thickness based on needle width (*T*_mes.w_) (B) and fraction of mesophyll parenchyma to total needle area (*f*_mes_) (C) of four evergreen conifer species (*Picea glehnii*: *Pg*, *P. jezoensis*: *Pj*, *Abies sachalinensis*: *As*, and *Taxus cuspidata*: *Tc*) grown under open (left, open bars) and shade conditions (right, closed bars). Different letters indicate significant differences among means of the combination of species and light conditions at *P* < 0.05. Values are means + s.e. (*n* = 4).

**Fig. 10.**
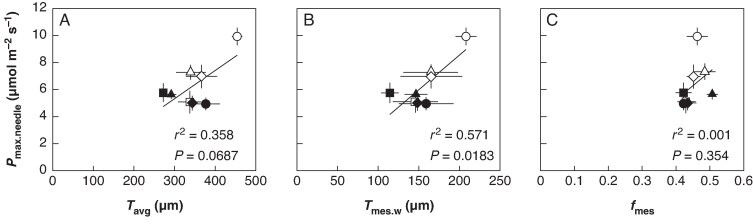
Relationships between the needle-level maximum photosynthetic rate (*P*_max.needle_) and the average needle thickness (*T*_avg_) (A), average mesophyll thickness (*T*_mes.w_) (B) and fraction of mesophyll parenchyma (*f*_mes_) (C) in four conifer species (*Picea glehnii*: triangles, *P. jezoensis*: diamonds, *Abies sachalinensis*: rectangles, and *Taxus cuspidata*: circles) grown under open (open symbols) and shade (closed symbols) conditions. Linear regression analysis was conducted for the pooled data across light conditions (solid line).

Significantly higher average needle thickness (*T*_avg_) was observed in open-grown *T. cuspidata* than in shade-grown *P. glehnii* and *A. sachalinensis* (Figs. 8 and 9A). Similarly, open-grown *T. cuspidata* showed higher average mesophyll thickness (*T*_mes.w_) than shade-grown *A. sachalinensis* (Fig. 9B). Regarding the fraction of mesophyll tissue to total needle cross-sectional area (*f*_mes_), there were no significant differences among the combinations of species and light conditions (Fig. 9C). Based on *t*-tests additionally conducted for each species to assess the effects of light treatment on these three parameters, a significant difference in *T*_mes.w_ was found only between open- and shade-grown *T. cuspidata* (*P* = 0.0496). Among the relationships between these three parameters and needle-level photosynthetic rate (*P*_max.needle_) (Fig. 10), a statistically significant increasing trend was observed only between *T*_mes.w_ and *P*_max.needle_.

## DISCUSSION

Needle configuration along a shoot appears to be closely associated with shade tolerance among the four conifer species native to northern Japan. More shade-tolerant species tend to have less densely packed needles, as indicated by a lower *A*_T_/*A*_P_ ratio, which may contribute to increased light reception at the needle surface (Niinemets, 1997, 2010). Furthermore, shade-grown seedlings had less-packed needles than open-grown counterparts, irrespective of species, suggesting shade-acclimation via shoot morphological changes to intercept more light at the needle surface. Shoot-level *P*_max_ (*P*_max.shoot_) was positively related to *A*_T_/*A*_P_ in all species across light conditions (Fig. 3B). Thus, shoot morphology should play a functional role for acclimating to growing light environments in these conifer species, similar to the changes in leaf thickness of broadleaved trees along within-canopy light gradients (Oguchi *et al*., 2005; Kitao *et al*., 2006, 2012; Aneja *et al*., 2025).

Although less-packed needles (i.e. lower *A*_T_/*A*_P_) provide an advantage for light interception under shade, they might increase the risk of photoinhibition under strong light conditions, such as transient sun flecks (Long *et al*., 1994). In this context, a shade-intolerant pioneer species, *P. glehnii*, might have higher capacity to avoid photoinhibition by mutual shading of densely packed needles within a shoot. Notably, there was no difference in *P*_max.needle_ in *P. glehnii* between open and shade conditions, as observed in *P. jezoensis* and *A. sachalinensis*. Thus, shoot-level photosynthetic acclimation to strong light in these species, evidenced by higher *P*_max.shoot_ in open-grown seedlings compared to shade-grown seedlings, may be primarily achieved by morphological changes of shoot needle packing. Conversely, *T. cuspidata*, a highly shade-tolerant species, increased its needle-level photosynthetic capacity (*P*_max.needle_) under the open condition. This phenomenon can be attributed to the insufficient mutual shading of the least densely packed needles (characterized by the lowest *A*_T_/*A*_P_), which is compensated for by an increase in *P*_max.needle_, enabling *T. cuspidata* to efficiently consume light energy through photosynthesis and thereby mitigate the risk of photoinhibition (Murchie and Niyogi, 2011; Kitao *et al*., 2018*b*, 2019*b*). The combination of less-packed needles and no enhancement in *P*_max.needle_ in open-grown seedlings of *A. sachalinensis* might cause photoinhibition, indicated by the lower *F*_v_/*F*_m_ (Krause, 1994; Werner *et al*., 2002; Kitao *et al*., 2022). This also suggests that *A. sachalinensis* might be a shade-specialist with a limited capacity to acclimate to full sunlight (Nakagawa *et al*., 2001, 2017; Kitao *et al*., 2018*a*, 2024).

Needle packing within shoots reduced the light intensity reaching needle surfaces (Figs 2B and 3A). Even moderate attenuation, such as the τ values around 0.4 observed in *P. glehnii*, *P. jezoensis* and *A. sachalinensis*, may be sufficient to support shade-acclimated needle traits under open conditions, given that leaf morphological plasticity tends to plateau under deeper shade (Kitao *et al*., 2006). While mutual shading probably contributes to this effect, the extent of morphological adjustment in response to light may be inherently constrained, as indicated by needle anatomical analyses. The conservative leaf morphology typical of conifer species such as *P. glehnii*, *P. jezoensis* and *A. sachalinensis* may therefore also help maintain shade-like photosynthetic traits under open conditions, in concert with mutual shading effects (Oguchi *et al*., 2003, 2005; Retta *et al*., 2024).

A novel finding is that *P. glehnii*, *P. jezoensis* and *A. sachalinensis* developed open-grown needles with mostly the same traits (*P*_max_, and ϕ) as shade-grown needles (Fig. 5). Higher LCP_needle_ observed in open-grown seedlings reflected higher respiration rate with higher LMA than in shade-grown seedlings. However, increased LMA and N_area_ do not appear to markedly contribute to increasing photosynthetic capacity (Fig. 6), suggesting that biomass and N might be allocated into structural components rather than into photosynthetic components in these species, thereby contributing to their extended leaf lifespan (Takashima *et al*., 2004; Onoda *et al*., 2017; Hikosaka *et al*., 2025). The values of LCP_needle_ in open-grown needles (≈20 µmol m^−2^ s^−1^) were comparable to those in seedlings of shade-tolerant broadleaved species, *Acer mono* and *Quercus mongolica* var. *crispula*, grown under shade (10 % of full sunlight: same as in the present study) (Kitao *et al*., 2016). According to a broad assessment of shade tolerance among 440 native woody species in Taiwan (Kuo *et al*., 2021), an LCP_needle_ of ≈20 µmol m^−2^ s^−1^ corresponds to a moderate level of shade tolerance. However, age-dependent photosynthetic traits in needles of *Abies alba* showed that net photosynthetic rate declined with age, whereas quantum yield of photochemistry of PSII (ΦPSII) at low light intensity (69 µmol m^−2^ s^−1^) remained stable up to 6 years (Robakowski and Bielinis, 2017). Similarly, respiration rate in Scots pine (*Pinus sylvestris* L.) needles declined steeply from current-year to older needles (Zha *et al*., 2002). As ΦPSII and the initial slope of the light response curve (ϕ) are linearly correlated (Werner *et al*., 2001), LCP in open-grown needles may decrease with age, indicating increased shade tolerance due to reduced respiration and sustained photochemical efficiency. Further investigation is needed to validate this possibility in field-grown conifer trees.

Shade-needles developed under full sunlight might not necessarily be a general trait among evergreen conifer trees, as was suggested for *T. cuspidata*, where higher *P*_max_ was observed in open-developed needles. *Taxus cuspidata* is known to develop adventitious branches, and the shape of the tree becomes shrub-like. This species may develop new shoots under shade within the canopy, which have high shade tolerance with less-packed needles and low *P*_max_, achieving efficient carbon gain as a whole canopy via an optimum distribution of nitrogen along the canopy light gradient (Niinemets, 2007; Hikosaka, 2014; Kitao *et al*., 2018*b*). Further studies are needed to investigate species-specific responses to different light environments from the viewpoint of shoot- and needle-level photosynthetic traits and needle configuration along a shoot. In particular, shade-intolerant pioneer species with less-densely packed needle arrangements, such as *Pinus sylvestris* (Niinemets, 2010) and *Pinus ponderosa*, which has shown a positive relationship between *P*_max.needle_ and LMA (Bond *et al*., 1999), are expected to employ different strategies for light acclimation of photosynthesis during their needle lifespan compared to the four species investigated in the present study.

A gradual decrease in needle packing, resulting from increased spacing between needles due to age-dependent stem thickening, may enhance light interception at the needle surface under the shade conditions of the canopy interior (Niinemets, 2007, 2010). In addition, a higher proportion of diffuse light within the canopy can mitigate mutual shading among needles (Carter and Smith, 1985). These structural and optical factors, together with the inherently shade-characterized photosynthetic traits of the needles, may enable shoots to sustain positive carbon assimilation throughout their lifespan. Needle longevity may also contribute to nitrogen retention in older shoots, keeping it accessible for redistribution to younger shoots (Wyka *et al*., 2016), thereby supporting acclimation to fluctuating environmental conditions. This buffering role of nitrogen storage parallels the function of carbohydrate reserves in promoting stress resilience and metabolic flexibility (Thalmann and Santelia, 2017).

## CONCLUSION

In *P. glehnii*, *P. jezoensis* and *A. sachalinensis*, shade-characteristic needles developed even under full sunlight, probably due to mutual shading from dense needle packing and/or conservative leaf anatomy. In these species, shoot-level light acclimation may be regulated by needle configuration, consistent with species-specific shade tolerance. Higher needle density was linked to greater shoot-level photosynthetic capacity under open conditions. In contrast, *T. cuspidata* showed increased needle-level photosynthetic capacity and leaf morphological changes, possibly compensating for sparse needle packing. These findings highlight needle packing as a key axis of light acclimation in evergreen conifers. The persistence of shade-characteristic needles, along with reduced packing from age-related stem thickening, may support needle longevity under canopy shading.

## Supplementary Material

mcag030_Supplementary_Data

## SUPPLEMENTARY INFORMATION

Supplementary data are available at *Annals of Botany* online. Discussion. Influence of wound respiration on the estimation of needle respiration. Figure S1. Procedure of gas exchange measurements determining shoot- and needle-level photosynthetic rates. Figure S2. Transverse sections of the needles from all individuals of *P. glehnii* grown under open and shade conditions. Figure S3. Transverse sections of the needles from all individuals of *P. jezoensis* grown under open and shade conditions. Figure S4. Transverse sections of the needles from all individuals of *A. sachalinensis* grown under open and shade conditions. Figure S5. Transverse sections of the needles from all individuals of *T. cuspidata* grown under open and shade conditions. Figure S6. Relationship between LMA and needle respiration rate in needles of four evergreen conifer species grown under open and shade conditions.

## AI ASSISTANCE

In the writing process, the authors used *Microsoft Copilot* solely for English editing and language refinement. The AI tool was utilized under the authors’ oversight, who carefully reviewed and edited the outcome, assuming full responsibility for the article’s final content and conclusions.
