# Supplementary material for: Densely packed needles along the shoots of evergreen conifers exhibit shade-acclimated photosynthetic characteristics even under full sunlight
Source: Ann Bot. 2026 Apr 8;137(7):2399–411. doi: 10.1093/aob/mcag030 (PMC13319511; doi:10.1093/aob/mcag030)
Supplement: mcag030_Supplementary_Data [file mcag030_supplementary_data.zip › Supplemental_Discussion.docx]

**Supplemental Discussion**

*Influence of wound respiration on the estimation of needle respiration*

Respiration rates of a shoot were measured under three conditions: 1) with all needles, 2) with a single layer of needles, and 3) without needles (cf. Fig. S1 in Supporting Information). In all cases, the total respiration rate of the shoot (R) can be expressed as:

R = *a* × [stem dry mass] + *b* × [attached needle dry mass] + *c* × [detached needle dry mass],

where:

- *a* is the dry-mass-based respiration rate of the stem (µmol g^-1^ s^-1^),
- *b* is the dry-mass-based respiration rate of the needles (µmol g^-1^ s^-1^), and
- *c* is the wound respiration rate induced by needle detachment, assumed to be proportional to the detached needle dry mass (µmol g^-1^ s^-1^).

The coefficients (*a*, *b*, and *c*) were estimated using a multiple linear regression analysis with the intercept set to zero. This analysis was performed with the *lm* function in R (version 6_2.6.0) using pooled data from open- and shade-grown seedlings of four conifer species (*P. glehnii*, *P. jezoensis*, *A. sachalinensis*, and *T. cuspidata*).

The results of the regression analysis are summarized below (Table SD1):

**Table SD1.** Summary of multiple linear regression for shoot respiration rate as a function of stem, attached needle, and detached needle dry mass

| Dependent variable |  | *R*^2^ | *p*-value |  | Independent variable | coefficient | *p*-value |
| --- | --- | --- | --- | --- | --- | --- | --- |
| Shoot respiration rate |  | 0.969 | < 2.2e-16 |  | Stem dry mass | 48.79 | 3.25e-07 |
|  |  |  |  |  | Attached needle dry mass | 48.54 | < 2e-16 |
|  |  |  |  |  | Detached needle dry mass | 1.92 | 0.462 |

Shoot respiration rates were effectively estimated (Fig. SD1) by the equation:

R = 48.79 × [stem dry mass] + 48.54 x [attached needle dry mass] + 1.92 x [detached needle dry mass].

The coefficient for detached needle dry mass (1.92) was relatively small, accounting for only 3.95% of the coefficient for attached needle dry mass (48.54). Therefore, the influence of wound respiration on the estimation of needle respiration can be considered negligible.


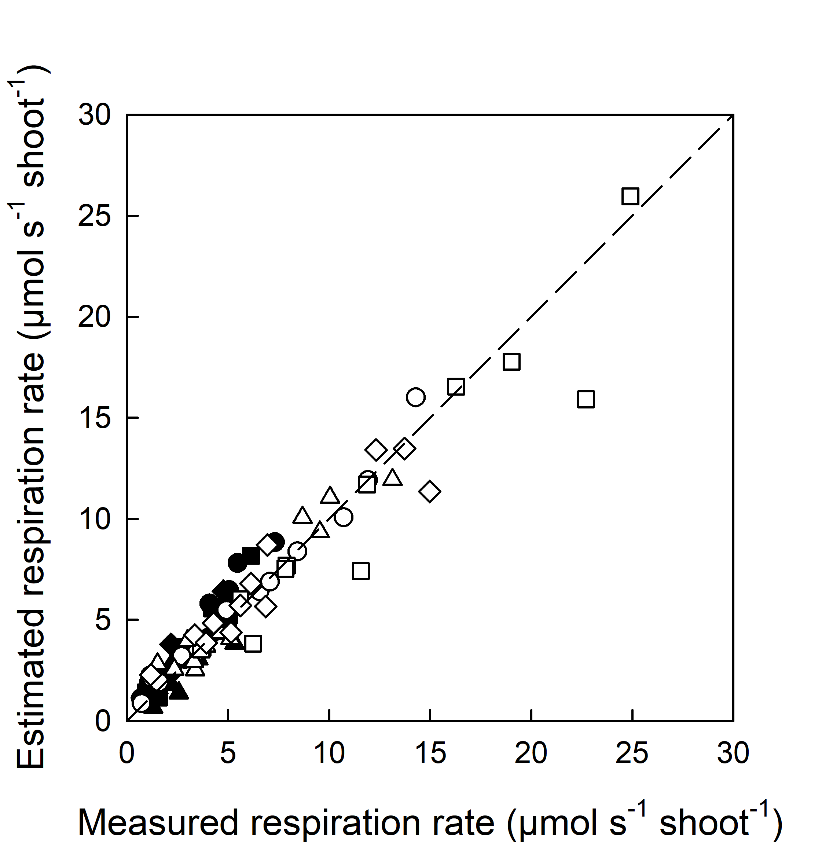


**Figure SD1** Relationship between measured and estimated respiration rates in shoots under three conditions: without needles, with a single layer of needles, and with all needles. The data includes open-grown seedlings (open symbols) and shade-grown seedlings (closed symbols) of four conifer species—*P. glehnii* (triangles), *P. jezoensis* (diamonds), *A. sachalinensis* (rectangles), and *T. cuspidata* (circles). Shoot respiration rates (R) were estimated using the equation: R = 48.79 × [stem dry mass] + 48.54 × [attached needle dry mass] + 1.92 × [detached needle dry mass]. The dashed line represents the 1:1 line, which passes through the origin.
