# Supplementary material for: Densely packed needles along the shoots of evergreen conifers exhibit shade-acclimated photosynthetic characteristics even under full sunlight
Source: Ann Bot. 2026 Apr 8;137(7):2399–411. doi: 10.1093/aob/mcag030 (PMC13319511; doi:10.1093/aob/mcag030)
Supplement: mcag030_Supplementary_Data [file mcag030_supplementary_data.zip › Supplemental_Figures.rev.docx]

**Supporting information**


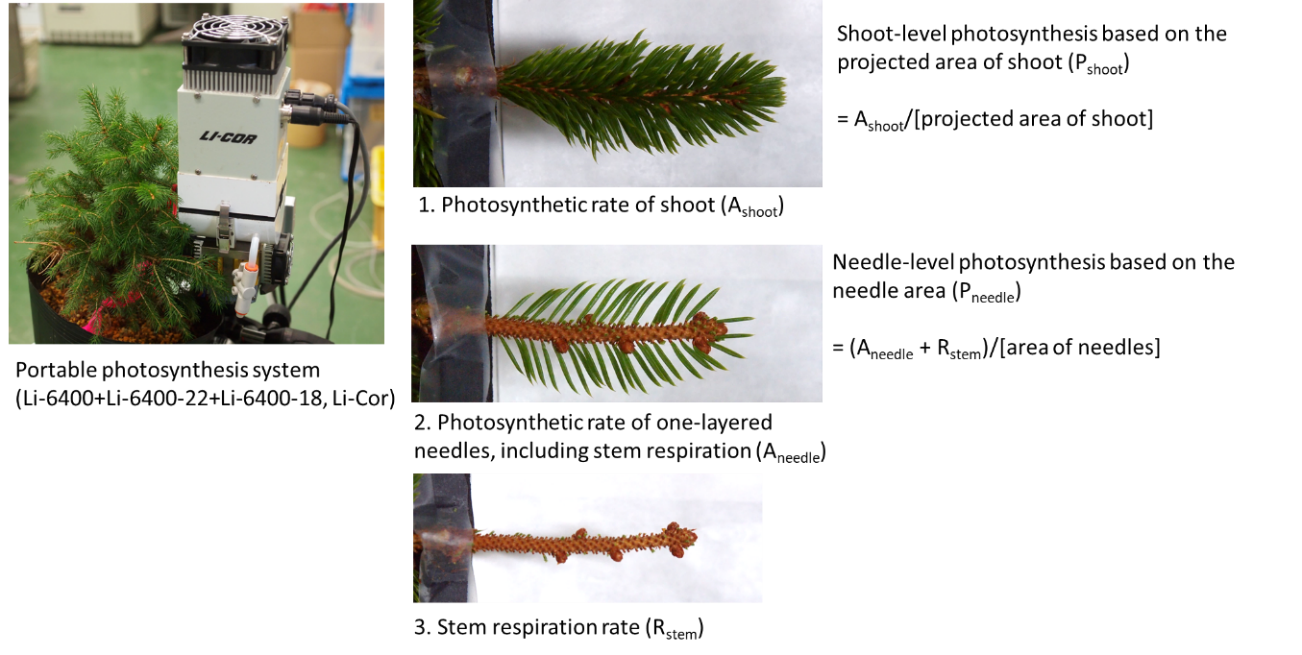


**Figure S1.** Procedure of gas exchange measurements determining shoot-level and needle-level photosynthetic rates. Projected shoot area and total needle area were calculated by using an image processing software (LIA32, ver 0.377e, Kazukiyo Yamamoto, 2004, https://www.agr.nagoya-u.ac.jp/~shinkan/LIA32/index-e.html).

**
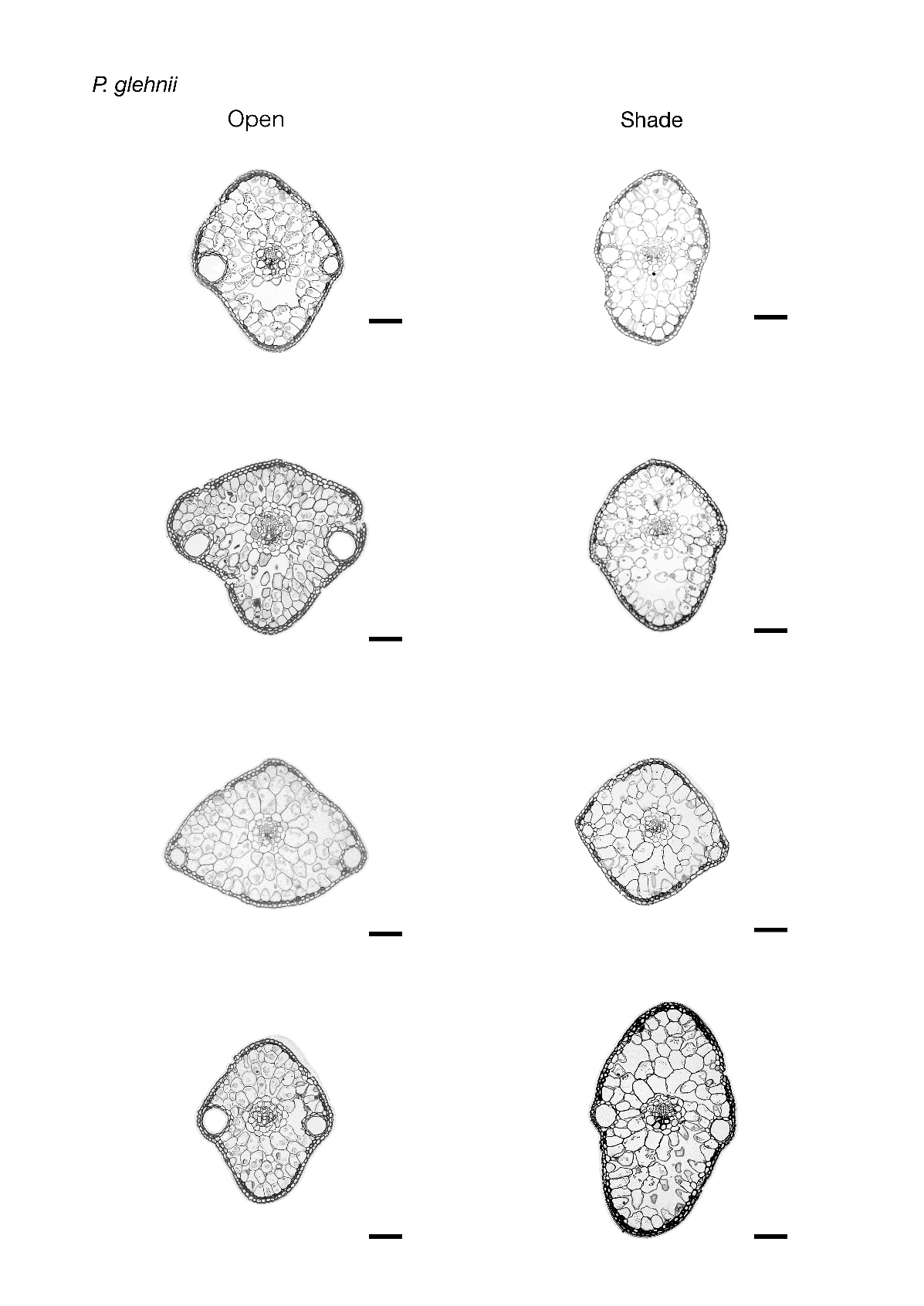
**

**Figure S2.** Transverse sections of the needles from all individuals of *P. glehnii* grown under open (left panel) and shade (right panel) conditions. All images were acquired using a 10× objective lens. Bars = 100 µm.


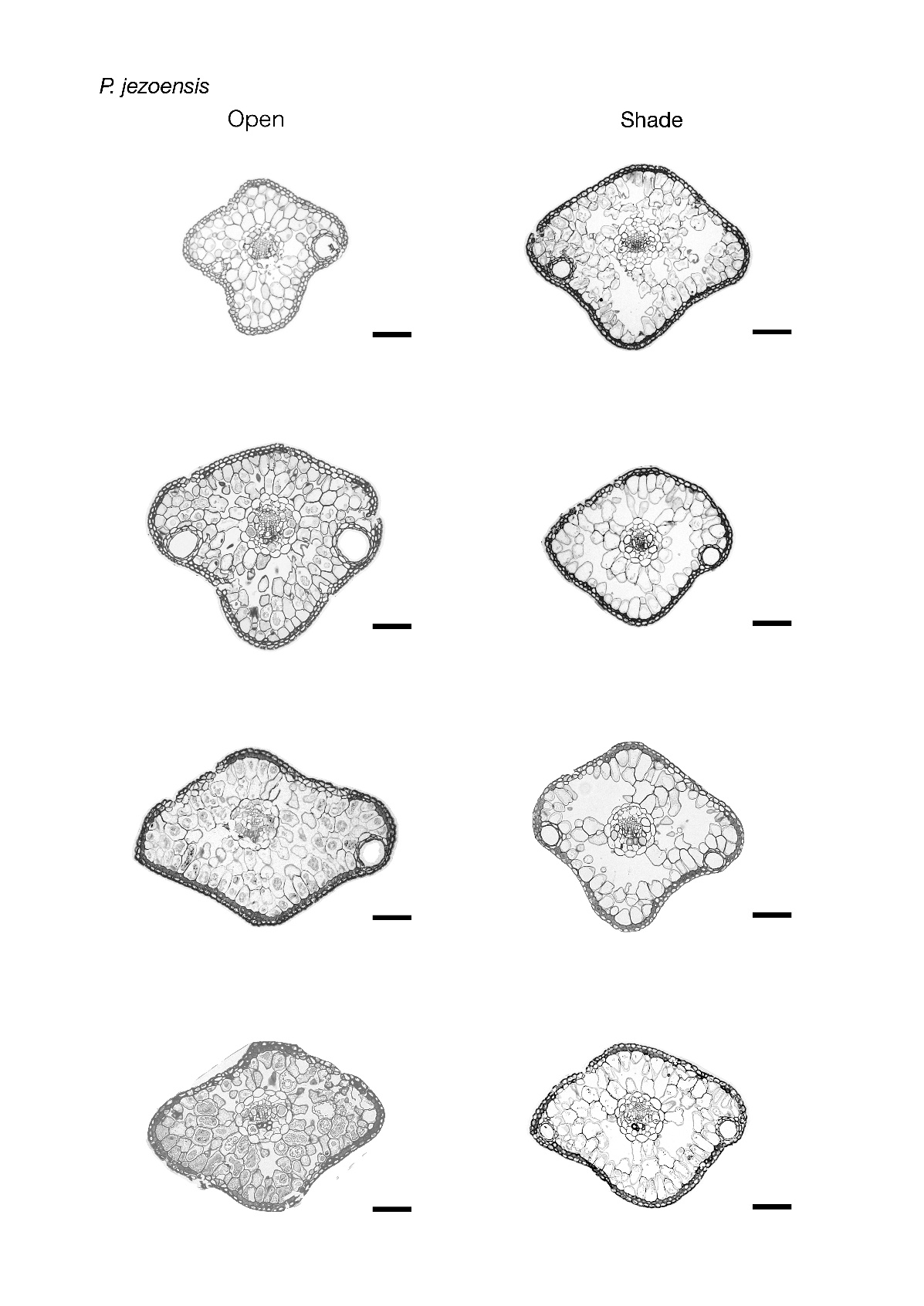


**Figure S3.** Transverse sections of the needles from all individuals of *P. jezoensis* grown under open (left panel) and shade (right panel) conditions. All images were acquired using a 10× objective lens. Bars = 100 µm.


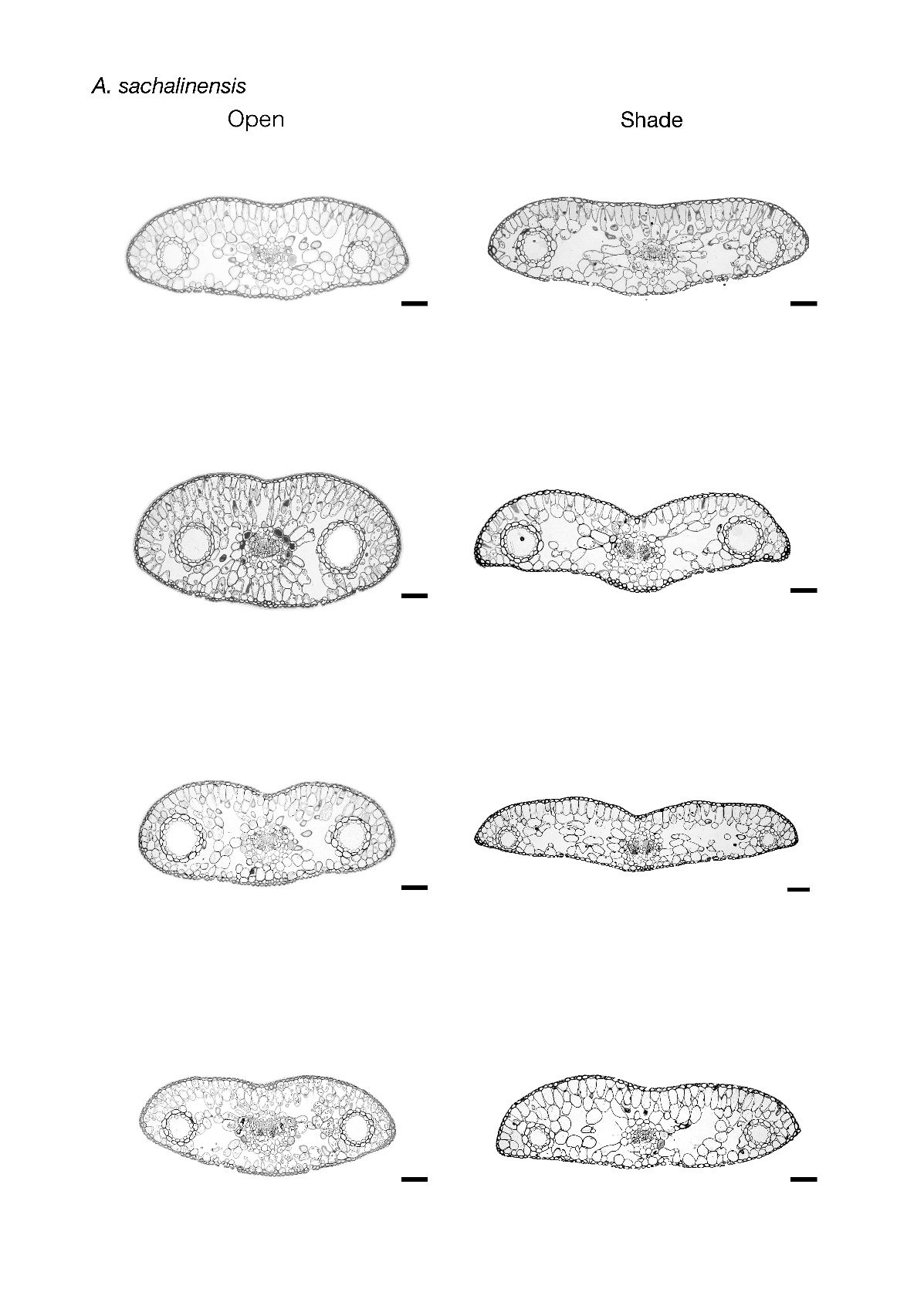


**Figure S4.** Transverse sections of the needles from all individuals of *A. sachalinensis* grown under open (left panel) and shade (right panel) conditions. All images were acquired using a 10× objective lens. Bars = 100 µm.

**
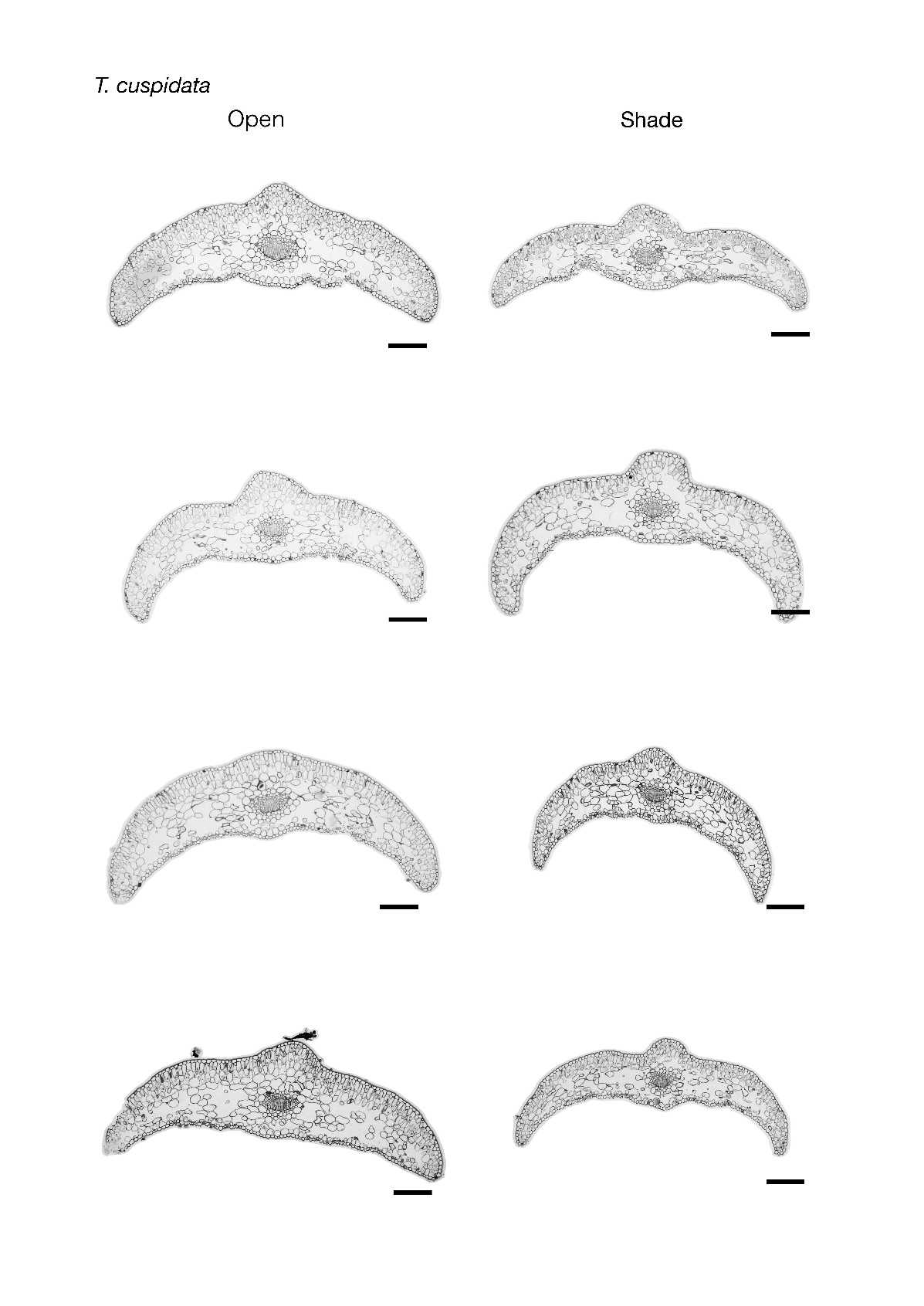
**

**Figure S5.** Transverse sections of the needles from all individuals of *T. cuspidata* grown under open (left panel) and shade (right panel) conditions. All images were acquired using a 4× objective lens. Bars = 250 µm.


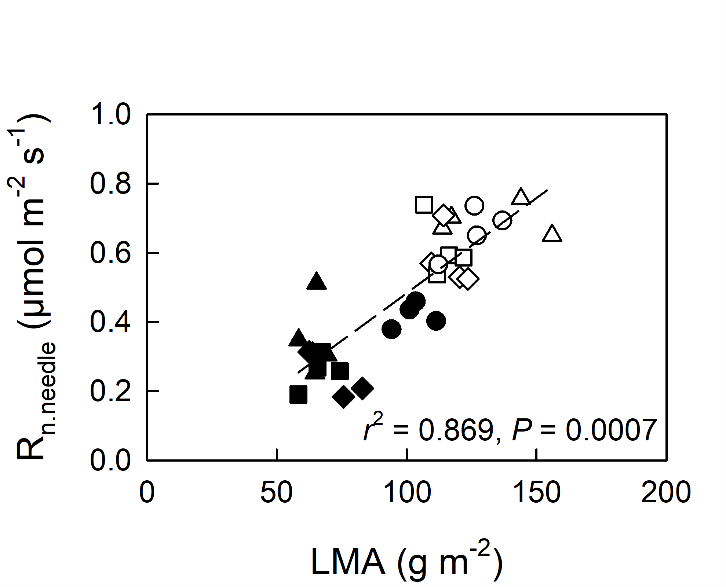


**Figure S6.** Relationship between LMA and needle respiration rate in needles of four evergreen conifer species (*P. glehnii*: triangles, *P. jezoensis*: diamonds, *A. sachalinensis*: rectangles, and *T. cuspidata*: circles) grown under open (open symbols) and shade (closed symbols) conditions. Linear regression (dashed line) was conducted for all the data pooled across species and light conditions.
